# Association of Sleep Duration with Chronic Diseases in the European Prospective Investigation into Cancer and Nutrition (EPIC)-Potsdam Study

**DOI:** 10.1371/journal.pone.0030972

**Published:** 2012-01-25

**Authors:** Anne von Ruesten, Cornelia Weikert, Ingo Fietze, Heiner Boeing

**Affiliations:** 1 Department of Epidemiology, German Institute of Human Nutrition Potsdam-Rehbruecke, Nuthetal, Germany; 2 Department of Cardiology and Angiology, Center of Sleep Medicine, CCM, Charité – Universitätsmedizin Berlin, Berlin, Germany; Cardiff University, United Kingdom

## Abstract

**Background:**

In view of the reduced number of hours devoted to sleep in modern western societies the question arises what effects might result from sleep duration on occurrence of chronic diseases.

**Methods:**

Data from 23 620 middle-aged participants of the European Prospective Investigation into Cancer and Nutrition (EPIC)-Potsdam study, that were recruited between 1994–1998, were analyzed by using Cox proportional hazard regression to examine the association between self-reported sleep duration at baseline and incidence of chronic diseases, such as diabetes, myocardial infarction, stroke, and cancer.

**Results:**

During a mean follow-up period of 7.8 years 841 incident cases of type 2 diabetes, 197 cases of myocardial infarction, 169 incident strokes, and 846 tumor cases were observed. Compared to persons sleeping 7-<8 h/day, participants with sleep duration of <6 h had a significantly increased risk of stroke (Hazard Ratio (HR) = 2.06, 95% confidence interval (CI): 1.18–3.59), cancer (HR = 1.43, 95% CI: 1.09–1.87), and overall chronic diseases (HR = 1.31, 95% CI: 1.10–1.55) in multivariable adjusted models. Self-reported daytime sleep at baseline was not associated with incident chronic diseases in the overall study sample. However, there had been an effect modification of daytime sleep by hypertension showing that daytime sleep was inversely related to chronic disease risk among non-hypertensive participants but directly related to chronic diseases among hypertensives.

**Conclusion:**

Sleep duration of less than 6 h is a risky behavior for the development of chronic diseases, particularly stroke and cancer, and should be therefore addressed in public health campaigns.

## Introduction

The examination of the relationship between sleep and health has gained increasing scientific importance, especially in population-based studies. In particular, it has been suggested that sleep is essential for recreation, energy conservation, repair and infection control, and that it is important for the programming of the brain [1], [2], [3].

Understanding the importance of sleep is particularly relevant as sleeping habits have been changing since the industrial age. In our modern 24-hour a day society, the overall prevalence of sleep disorders and daytime sleepiness is high and duration of sleep per day tends to be decreased [3], [4], [5], [6]. This trend is a result of less dependency of daylight hours, increased shift-work, long working hours [6], [7], [8], increased time spent commuting to and from the workplace [7], and alterations in lifestyle. Alterations in lifestyle may for example refer to the double burden of taking care of family's needs and work-related responsibilities [8]. The 2005 Sleep in America poll indicates that, on average, adults in America are sleeping 6.9 h a night when considering both weekday and weekend sleep. 40% of the respondents reported getting less than 7 h of sleep per night on weekdays. A large majority (75%) reported having had at least one symptom of a sleep problem, e.g. difficulties falling asleep, difficulties maintaining sleep, sleep-related breathing disorders, or getting up feeling not rested, a few nights a week or more within the past year [9].

Due to the adverse changes in sleeping habits the question arises which long-term health consequences might result from sleep deficiency. Emerging data suggest that sleep deprivation has major metabolic and cardiovascular consequences and consequently might be a risk factor for poor health in the future [3], [10], [11], [12], [13]. Habitual sleep durations of ≤5–6 h sleep per 24 h may lead to an increased risk of incident diabetes [14], [15], [16], [17], [18], cardiovascular diseases [19], [20], [21], [22], [23], and breast cancer [24]. On the other hand, in some studies, also among long sleepers (>8–9 h) the risk for incident diabetes [15], [16], [25], [26] and cardiovascular diseases [19], [21], [27] was elevated. In addition, also daytime naps are discussed to have detrimental effects on health, e.g. on diabetes [17] or cardiovascular risk [28]. Up to now, just a few studies concerning this topic were conducted in Europe and in general these studies focused on one endpoint and thus did not take into account competing risks for different chronic disease endpoints.

Therefore, this analysis primarily aims to investigate whether sleeping habits practiced nowadays are related to the occurrence of the most important chronic diseases, i.e. the combination of incident type 2 diabetes, cardiovascular diseases, and cancer, within the European Prospective Investigation into Cancer and Nutrition (EPIC)-Potsdam study.

## Materials and Methods

### Study population: the EPIC-Potsdam study

The European Prospective Investigation into Cancer and Nutrition (EPIC) study is an ongoing large-scale cohort study on diet and chronic diseases, especially cancer, with over half a million participants across Europe. EPIC-Potsdam is one out of 23 centers of EPIC with 27 548 participants aged mainly 35–65 years that had been recruited between 1994 and 1998 from the general population of Potsdam and adjacent communities. The study instruments of the baseline examination included a computer-guided interview on lifestyle habits and medical history, self-administered questionnaires on food consumption and lifestyle as well as physical examinations performed by trained staff at the study centre [29]. The EPIC-Potsdam study has been approved by the Ethical Committee of the Federal State of Brandenburg. Written informed consent for measurements and inquiries including prospective data collection has been obtained from all participants before joining EPIC-Potsdam.

Every 2–3 years the participants received a mailed follow-up questionnaire to assess incident diseases, medication, and changes in diet and other lifestyle factors. Tracing of non-responders, reminder services, and computer programs controlling for completeness or implausible values contribute to the generation of valid and complete follow-up data and high response rates for each follow-up round (93–96%) [30].

Participants with prevalent diabetes mellitus, myocardial infarction, stroke, or cancer (all except non-melanoma skin cancer) (n = 3 130), missing follow-up time (n = 589), missing data on exposure or relevant covariates (n = 207) as well as those reporting not to sleep at all (n = 2) were excluded. Thus, 23 620 participants (14 497 women and 9 123 men) remained for the analyses. Study participants were followed up until 2007. The follow-up time (person-years) was derived from the age at recruitment and the age at one of the following end-points: date of diagnoses, date of death, or return of the last follow-up questionnaire, whichever came first.

### Assessment of exposure

Participants were asked about their average duration of sleep (in hours) at night and at day during the last 12 months with two open questions in the baseline interview. Moreover, also sleep disorders were assessed in the baseline interview with the following questions: “Have you ever suffered from sleep disorders that were treated by a physician?” If this question was answered with “yes”, participants were further asked: “How long did you suffer from sleep disorders? Please total all periods in which you suffered from sleep disorders.”

### Assessment of chronic diseases

Information about potential incident chronic diseases occurring during follow-up were regularly assessed from self-reports of the respective condition, medication use, or reasons for a reported change in diet in the follow-up questionnaires. All potential incident cases were verified using information of medical reports from the treating physician, cancer registries, or death certificates [30]. Diseases were coded based on the International Classification of Disease (ICD-10: I21 for myocardial infarction, I60, I61, I63, I64 for stroke, E11 for diabetes, and C00-C97 for cancer (except C44: non-melanoma skin cancer). In case of multiple diseases, only the first clinical event was considered for the analysis.

### Statistical analyses

Sleep duration at baseline was considered as the main exposure variable. Based on the habitual sleep duration, participants are classified as short, long, or normal sleepers. Therefore, the sleep duration at night and at day was summed and participants were categorized into five groups according to their sleep duration following the distribution of sleep duration in this cohort and cut-offs used in former publications: <6, 6-<7, 7-<8, 8-<9 and ≥9 h per 24 h (<6 h is defined as short, 6-<7 h as shortened, 7-<8 h as normal/average, 8-<9 h as normal/prolonged and ≥9 h as long sleep duration). These groups were characterized by descriptive univariate statistics in order to examine the correlates of long and short sleep. Baseline characteristics of the study population are presented as arithmetic means and standard deviations (for continuous variables) and percentages (for categorical variables).

The risk of developing a chronic disease in different groups of sleep duration was analyzed by means of Cox proportional hazard regression analyses. Age- and sex- as well as multivariable-adjusted hazard ratios (HR) and 95% confidence intervals (CI) were calculated using 7-<8 h of sleep as the reference group. This reference was chosen for two reasons. First, 7 h of sleep had been shown to be associated with the lowest risk of mortality [31], [32], [33] and also morbidity e.g. cardiovascular disease [13] or diabetes [12]. Second, the average sleep duration was 7 h in this sample (mean: 7.3 h, median: 7 h).

The primary endpoint was defined as the first incident chronic disease – i.e. type 2 diabetes, myocardial infarction, stroke, or cancer. Additional events at a later date were not considered. The follow-up time was defined as time period between the age of recruitment and the age of exit (age of diagnoses, death, or censoring). To be less sensitive against violation of the proportional hazards assumption, all models were stratified by age. Relevant covariates, e.g. socioeconomic and lifestyle factors were considered by multivariable adjustment. Therefore, four models were carried out. The first model (model 1, reduced model) was adjusted for sex. The second model (model 2) additionally included sleeping disorders (yes/no), alcohol intake from beverages (non-consumers; men: 1. >0–12 g/d, 2. >12–24 g/d, 3. >24 g/d; women: 1. >0–6 g/d, 2. >6–12 g/d, 3. >12 g/d), smoking status (never, former, current), walking, cycling, sports (hours/week), employment status (employed vs. unemployed), and education (university of applied sciences or university degree vs. technical school or lower degree). Model 3 was further adjusted for potential intermediates, i.e. BMI (kg/m^2^), waist-to-hip ratio, prevalent hypertension at baseline (yes/no), and history of high blood lipid levels at baseline (yes/no). Model 4 furthermore included consumption of caffeinated beverages (coffee and black tea in g/day), satisfaction with life (4 levels: very satisfied, rather satisfied, rather dissatisfied, very dissatisfied), satisfaction with health (4 levels: very satisfied, rather satisfied, rather dissatisfied, very dissatisfied), and intake of antidepressants (yes/no). Covariables were defined based on *a priori* knowledge on risk factors for the investigated outcomes. In general, the same covariates were included in each model, except baseline prevalent hypertension and higher blood lipid levels, which were not included in the multivariable models for cancer.

In addition to sleep duration, we further investigated the effect of timing of sleep (daytime naps (yes/no) and night-time sleep (<6, 6-<7, 7-<8, 8-<9 and ≥9 h)) on the risk of chronic diseases using analogous multivariable Cox regression models.

Moreover, interactions of sleep duration (continuous) with sex, sleeping disorders (yes/no), age (<60 vs. ≥60 years), alcohol intake (category 2 and 3 vs. category 1), obesity (BMI ≥30 kg/m^2^: yes/no), and hypertension (yes/no) were investigated. Furthermore, interactions of these variables with daytime sleep were examined. Therefore, models with and without interaction terms were compared using the likelihood-ratio test. All *p* values were two-sided and a significance level of p<0.05 was applied.

All analyses were performed with SAS statistical software (release 9.2; SAS Institute Inc, Cary, North Carolina).

## Results

The baseline characteristics of participants of the EPIC-Potsdam cohort according to sleep duration are described in **Table 1**. Older people, women, and individuals with less education were more likely to report long sleep durations (≥9 h). Of note, there was a relatively low proportion of employed persons among long sleepers (34% compared to 66–78% in the other groups) which is partly due to the relatively high amount of (early) retired persons in this group. Furthermore, also the group of short sleepers (<6 h) was slightly older and less high educated compared to average sleepers.

**Table 1:** Baseline Characteristics and Risk Factors for Chronic Disease by Self-reported Sleep Duration.

Sleep duration	<6 h/day	6-<7 h/day	7-<8 h/day	8-<9 h/day	≥9 h/day
No. of subjects: 23 620 (100%)	1 242 (5.3%)	4 653 (19.7%)	9 820 (41.6%)	6 133 (26.0%)	1 772 (7.5%)
Age (years)	50.9 (8.3)	48.8 (8.4)	48.5 (8.5)	49.4 (9.3)	53.9 (9.5)
Women, %	61.7	57.9	59.5	65.6	65.9
Employed, %	67.6	78.4	78.1	66.2	33.5
(Early) retirees, %	18.7	12.7	12.7	21.3	48.2
University of applied sciences/university degree, %	29.0	40.6	41.0	35.1	25.5
Sleeping disorders, %	26.7	15.8	12.8	12.6	15.8
Obesity (BMI ≥30 kg/m^2^), %	21.9	16.7	14.3	14.7	20.7
Prevalent hypertension, %	51.9	45.8	43.6	46.5	55.0
History of high blood lipid levels, %	32.4	24.8	23.3	24.9	32.5
Current smoker, %	25.4	23.8	19.5	18.4	21.6
Walking, cycling, sports (h/week)	11.5 (8.3)	10.4 (7.3)	10.4 (7.0)	11.0 (7.4)	12.2 (7.7)
Beverages, low-energy (g/day)^a^	1 279.9 (783.7)	1 197.4 (683.6)	1 109.8 (590.9)	1 118.9 (631.0)	1 165.4 (667.0)
Beverages, high-energy (g/day)^b^	248.2 (306.5)	245.0 (271.4)	232.3 (252.8)	241.7 (257.9)	252.8 (278.9)
Caffeinated beverages (g/day)^c^	600.6 (442.5)	584.9 (379.2)	545.2 (332.8)	521.5 (330.2)	513.7 (335.1)
Fresh fruit (g/day)	144.4 (99.1)	142.0 (96.5)	140.0 (94.1)	143.6 (97.7)	142.0 (99.6)
Vegetables (g/day)^d^	123.8 (61.9)	123.8 (59.3)	122.3 (61.0)	125.3 (59.5)	127.9 (62.1)
Whole grain bread and muesli (g/day)	50.9 (59.5)	53.2 (60.1)	50.2 (57.5)	50.7 (58.7)	46.5 (55.4)
Other bread and flakes (g/day)	133.1 (84.2)	137.8 (85.8)	130.6 (79.9)	131.7 (78.3)	124.1 (70.1)
Side dishes (g/day)^e^	94.1 (52.9)	95.6 (51.5)	97.0 (49.7)	100.6 (50.7)	109.7 (51.9)
Dairy products (g/day)^f^	254.5 (260.6)	236.9 (233.8)	228.2 (218.6)	229.8 (217.2)	239.5 (245.8)
Meat (g/day)^g^	114.8 (74.3)	116.8 (72.1)	116.5 (66.0)	112.2 (65.5)	109.6 (65.2)
Fish (g/day)	25.3 (31.1)	23.9 (23.7)	23.2 (24.0)	23.5 (26.5)	24.1 (23.3)
Eggs (g/day)	18.5 (18.1)	18.1 (18.2)	17.6 (15.6)	17.3 (14.1)	16.9 (16.8)
Butter (g/day)	8.1 (12.8)	9.3 (13.3)	8.7 (12.5)	9.0 (12.4)	9.1 (12.5)
Vegetable oil (g/day)	3.2 (3.2)	3.4 (3.3)	3.4 (3.4)	3.5 (3.5)	3.4 (3.2)
Sweets (g/day)^h^	117.5 (109.9)	114.7 (92.5)	113.9 (89.3)	112.4 (89.1)	117.5 (98.2)
Snacks (g/day)^i^	22.7 (20.3)	23.9 (20.5)	24.2 (19.8)	23.2 (18.8)	21.4 (18.6)
Alcohol intake (g/day)	13.7 (19.2)	14.6 (18.0)	14.6 (17.5)	13.8 (18.4)	13.5 (23.9)
Energy intake (kJ/day)	8 999.5 (3 252.3)	9 085.3 (3 050.2)	8 879.3 (2 802.2)	8 797.4 (2 806.7)	8 761.2 (2 962.1)
Very satisfied with life, %	17.1	21.2	23.4	27.1	28.3
Very satisfied with health, %	17.6	20.5	23.0	24.2	20.0
Intake of antidepressants, %	11.0	6.3	5.0	5.2	8.1

Categorical variables are presented as percentages within the respective subgroup and continuous variables are expressed as means with standard deviation in parentheses.

a Low-energy beverages: water, coffee (including de-caffeinated coffee), tea (including herbal tea), low-energy soft drinks (energy-reduced cola, lemonades).

b High-energy beverages: juice, high-energy soft drinks (cola, lemonades, non-alcoholic beer, malt beer).

c Caffeinated beverages: coffee, black tea.

d Vegetables are also including legumes.

e Side dishes: pasta, rice, potatoes.

f Dairy products: milk, yoghurt, curd, soured milk/kefir, cream, cheese.

g Meat: red meat, poultry, processed meat.

h Snacks: french fries, pizza, chips.

i Sweets: cakes, cookies, confectionary, sweet bread spread, desserts.

Many health variables showed an U-shaped relation with sleep duration, such as sleeping disorders, obesity, hypertension, and high blood lipid levels.

There were no marked differences in intake of important food groups between different sleep groups, except the intake of caffeinated beverages like coffee and black tea which is linearly inversely related to sleep duration. Therefore, food intake was not included into the multivariate models.

Satisfaction with life was linearly associated with length of sleep (17% were very satisfied with their life in persons sleeping <6 h/d compared to 28% in those sleeping ≥9 h/d). In contrast, U-shaped relations were observed for other psychosocial variables like satisfaction with health and use of antidepressants. The use of antidepressants was especially pronounced in persons sleeping <6 h/d (every 10^th^ person reported intake of antidepressants).

**Table 2** shows the association of sleep duration with risk of type 2 diabetes, myocardial infarction, stroke, and cancer. During a mean follow-up period of 7.8 years (184 388 person-years) 841 cases of incident diabetes, 197 cases of incident myocardial infarction, 169 cases of incident stroke, and 846 incident tumor cases occurred.

**Table 2:** Hazard ratios (HR) and 95% Confidence Intervals for Type 2 Diabetes, Myocardial Infarction, Stroke, and Cancer by Sleep Duration in the EPIC-Potsdam Cohort.

	<6 h/day	6-<7 h/day	7-<8 h/day	8-<9 h/day	≥9 h/day
***Overall chronic disease*** (cases/person-years)^a^	162/9 282	376/36 006	790/78 263	511/47 561	203/13 276
Crude rate per 1000 person-years	17.5	10.4	10.1	10.7	15.3
HR model 1^b^	1.54 (1.30–1.83)	1.02 (0.90–1.15)	1.0	1.00 (0.90–1.12)	1.09 (0.93–1.27)
HR multivariable-adjusted, model 2^c^	1.47 (1.24–1.74)	0.99 (0.88–1.13)	1.0	0.99 (0.88–1.11)	1.02 (0.87–1.20)
HR multivariable-adjusted, model 3^d^	1.33 (1.12–1.57)	0.96 (0.85–1.09)	1.0	0.97 (0.86–1.08)	0.97 (0.83–1.14)
HR multivariable-adjusted, model 4^e^	1.31 (1.10–1.55)	0.96 (0.85–1.09)	1.0	0.96 (0.86–1.08)	0.97 (0.83–1.14)
***Type 2 diabetes*** (cases/person-years)	62/9 282	161/36 006	330/78 263	199/47 561	89/13 276
Crude rate per 1000 person-years	6.7	4.5	4.2	4.2	6.7
HR model 1^b^	1.44 (1.10–1.89)	1.04 (0.86–1.25)	1.0	0.97 (0.81–1.16)	1.24 (0.97–1.58)
HR multivariable-adjusted, model 2^c^	1.36 (1.04–1.79)	1.02 (0.84–1.23)	1.0	0.95 (0.80–1.14)	1.16 (0.91–1.49)
HR multivariable-adjusted, model 3^d^	1.08 (0.82–1.42)	0.94 (0.78–1.14)	1.0	0.92 (0.77–1.10)	1.04 (0.81–1.32)
HR multivariable-adjusted, model 4^e^	1.06 (0.80–1.40)	0.94 (0.78–1.14)	1.0	0.92 (0.77–1.10)	1.05 (0.82–1.33)
***Myocardial infarction*** (cases/person-years)	18/9 282	33/36 006	81/78 263	44/47 561	21/13 276
Crude rate per 1000 person-years	1.9	0.9	1.0	0.9	1.6
HR model 1^b^	1.78 (1.07–2.97)	0.89 (0.59–1.34)	1.0	0.90 (0.62–1.30)	1.15 (0.70–1.89)
HR multivariable-adjusted, model 2^c^	1.54 (0.92–2.59)	0.83 (0.55–1.24)	1.0	0.85 (0.59–1.24)	0.97 (0.59–1.62)
HR multivariable-adjusted, model 3^d^	1.45 (0.87–2.44)	0.80 (0.53–1.20)	1.0	0.82 (0.56–1.19)	0.93 (0.56–1.55)
HR multivariable-adjusted, model 4^e^	1.44 (0.85–2.43)	0.80 (0.53–1.20)	1.0	0.82 (0.56–1.19)	0.89 (0.54–1.49)
***Stroke*** (cases/person-years)	17/9 282	29/36 006	55/78 263	44/47 561	24/13 276
Crude rate per 1000 person-years	1.8	0.8	0.7	0.9	1.8
HR model 1^b^	2.32 (1.34–4.01)	1.14 (0.73–1.79)	1.0	1.23 (0.83–1.84)	1.84 (1.12–3.02)
HR multivariable-adjusted, model 2^c^	2.20 (1.27–3.82)	1.15 (0.73–1.80)	1.0	1.19 (0.80–1.77)	1.69 (1.02–2.79)
HR multivariable-adjusted, model 3^d^	2.12 (1.22–3.68)	1.13 (0.72–1.77)	1.0	1.16 (0.78–1.73)	1.62 (0.98–2.68)
HR multivariable-adjusted, model 4^e^	2.06 (1.18–3.59)	1.13 (0.72–1.77)	1.0	1.16 (0.77–1.73)	1.65 (1.00–2.73)
***Cancer*** (cases/person-years)^f^	66/9 282	154/36 006	328/78 263	229/47 561	69/13 276
Crude rate per 1000 person-years	7.1	4.3	4.2	4.8	5.2
HR model 1^b^	1.46 (1.12–1.91)	1.00 (0.83–1.21)	1.0	1.04 (0.88–1.23)	0.82 (0.63–1.06)
HR multivariable-adjusted, model 2^c^	1.43 (1.10–1.87)	0.99 (0.82–1.20)	1.0	1.03 (0.87–1.23)	0.79 (0.61–1.04)
HR multivariable-adjusted, model 3^d^	1.43 (1.09–1.87)	0.99 (0.82–1.20)	1.0	1.03 (0.87–1.22)	0.79 (0.60–1.03)
HR multivariable-adjusted, model 4^e^	1.43 (1.09–1.87)	0.99 (0.82–1.20)	1.0	1.03 (0.87–1.22)	0.79 (0.60–1.03)

a Type 2 diabetes, myocardial infarction, stroke, or cancer, whichever occurs first.

b Stratified by age and adjusted for sex.

c Additionally adjusted for sleeping disorders (yes/no), alcohol intake from beverages (non-consumers, men: >0–12 g/d, >12–24 g/d, >24 g/d; women: >0–6 g/d, >6–12 g/d, >12 g/d), smoking status (never, former, current), walking, cycling, sports (hours/week), employment status (employed vs. unemployed), and education (technical school or lower degree vs. university of applied sciences or university degree).

d Adjusted for the variables in model 2 plus potential mediators: BMI (kg/m^2^), waist-to-hip ratio, prevalent hypertension at baseline (yes/no), and history of high blood lipid levels at baseline (yes/no).

e Adjusted for the variables in model 3 plus consumption of caffeinated beverages (coffee and tea in g/day), satisfaction with life (4 levels: very satisfied, rather satisfied, rather dissatisfied, very dissatisfied), satisfaction with health (4 levels: very satisfied, rather satisfied, rather dissatisfied, very dissatisfied), and intake of antidepressants (yes/no).

f Model 3–4 for cancer includes the same covariable-set as models for type 2 diabetes, myocardial infarction, and stroke, except prevalent hypertension at baseline (yes/no), and history of high blood lipid levels at baseline (yes/no).

**Figure 1:**
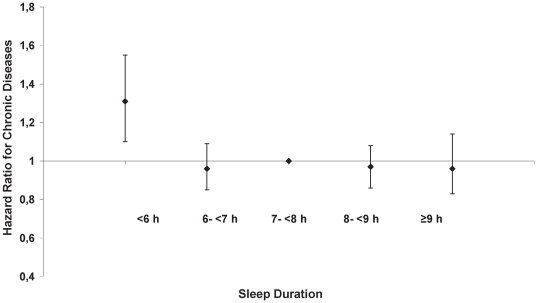
Association of sleep duration with overall chronic disease risk. Stratified by age and adjusted for sex, sleeping disorders, alcohol intake from beverages, smoking status, walking/cycling/sports, employment status, education, BMI, waist-to-hip ratio, prevalent hypertension at baseline, history of high blood lipid levels at baseline, consumption of caffeinated beverages, satisfaction with life, satisfaction with health, and intake of antidepressants.

Compared to persons with habitual sleep duration of 7-<8 h, participants reporting less than 6 h/day of sleep had an about 30% increased risk of developing overall chronic diseases in the fully adjusted model (model 4: HR = 1.31, 95% CI: 1.10–1.55) (**Figure 1** and **Table 2**).

Concerning specific disease outcomes, diabetes was related to short sleep, i.e. <6 h/day, in the model 1 as well as in model 2 (model 2: HR = 1.36, 95% CI: 1.04–1.79). Further inclusion of potential intermediates, predominantly BMI, attenuated the effect of sleep (model 3: HR = 1.08, 95% CI: 0.82–1.42). The association between short sleep and myocardial infarction was significant in the model 1 (HR = 1.78, 95% CI: 1.07–2.97) but inclusion of lifestyle factors, particularly smoking, attenuated this association (model 2: HR = 1.54, 95% CI: 0.92–2.59). In addition, short sleepers had a more than 2-fold increased risk of developing stroke compared to those sleeping 7-<8 h, independent of the kind of model chosen (model 4: HR = 2.06, 95% CI: 1.18–3.59). Short sleep duration is also an independent risk factor for overall cancer and consideration of several covariates did almost not change the effect estimate (model 4: HR = 1.43, 95% CI: 1.09–1.87). However, a competing risk analysis revealed that effect estimates for the single disease endpoints did not differ significantly from each other. Hence, it was possible to combine all events to one outcome called “overall chronic disease”.

Concerning long sleep duration, no effect was observed for the risk of chronic diseases in general, but it was shown to be associated with an increased risk of stroke (model 4: HR for ≥9 h/day = 1.65, 95% CI: 1.00–2.73).

We further studied the role of timing of sleep. Concerning overall chronic disease risk the effect estimates for sleep at night were similar to those of overall sleep duration because sleep at night constitutes a major part of overall sleep (<6 h: HR = 1.27 (1.08–1.50)) (**Table S1**). However, the association of <6 h of night-time sleep with stroke was only borderline significant after inclusion of further covariates and sleep duration at day into the multivariable regression model. This indicates that a slight compensation of short night-time sleep by daytime naps might be possible (HR = 1.66 (0.97–2.86)). Furthermore, the association of ≥9 h of night-time sleep with stroke was not significant (HR for ≥9 h of sleep at night: 1.56 (0.89–2.75)).

Only 11.5% of participants of the EPIC-Potsdam sample were reporting daytime sleep which had an average duration of ∼1 h (mean = 1.1 h). Therefore, only two exposure groups were compared: namely persons with and without daytime naps. The separate analysis of daytime naps revealed no influence on risk of chronic diseases in the overall study sample. The initially significant association of daytime napping with the risk of stroke in the model 1 (HR = 1.52 (1.04–2.24)) disappeared after multivariable adjustment (HR = 1.38 (0.93–2.03), table not shown). Interestingly, a significant interaction of daytime naps and hypertension in relation to chronic disease risk (p for interaction = 0.04) and cancer was observed (p for interaction = 0.03). Consequently, additional analyses that were stratified by prevalent hypertension were conducted (**Table 3**). Stratification by baseline hypertension revealed that in hypertensive persons that reported to sleep during the day the risk for overall chronic disease tends to be increased (HR = 1.13 (0.98–1.31)) which was mainly driven by stroke (HR = 1.51 (0.96–2.37)). In contrast, the risk of overall chronic disease is decreased among non-hypertensive persons reporting daytime naps (HR = 0.75 (0.59–0.96)) which is mainly due to a reduced risk for cancer (HR = 0.68 (0.49–0.95)).

**Table 3:** Hazard ratios (HR) and 95% confidence intervals for daytime sleep and chronic diseases stratified by prevalent hypertension.

	Non-hypertensive participants		Hypertensive participants	
Daytime sleep	No	Yes	No	Yes
	11 517 (90.4%)	1 223 (9.6%)	9 387 (86.3%)	1 493 (13.7%)
***Overall chronic disease*** (cases/person-years)^a^	604/91 920	85/9 618	1 080/71 959	273/10 890
Crude rate per 1000 person-years	6.6	8.8	15.0	25.1
HR model 1^b^	1.0	0.82 (0.65–1.05)	1.0	1.22 (1.06–1.41)
HR model 2^c^	1.0	0.77 (0.61–0.98)	1.0	1.21 (1.04–1.39)
HR model 3^d^	1.0	0.75 (0.59–0.96)	1.0	1.13 (0.98–1.31)
***Type 2 diabetes*** (cases/person-years)	178/91 920	24/9 618	521/71 959	118/10 890
Crude rate per 1000 person-years	1.9	2.5	7.2	10.8
HR model 1^b^	1.0	0.85 (0.54–1.33)	1.0	1.22 (0.99–1.51)
HR model 2^c^	1.0	0.81 (0.51–1.27)	1.0	1.21 (0.97–1.50)
HR model 3^d^	1.0	0.77 (0.49–1.22)	1.0	1.05 (0.84–1.30)
***Myocardial infarction*** (cases/person-years)	51/91 920	10/9 618	105/71 959	31/10 890
Crude rate per 1000 person-years	0.6	1.0	1.5	2.8
HR model 1^b^	1.0	1.20 (0.58–2.49)	1.0	1.41 (0.92–2.17)
HR model 2^c^	1.0	1.00 (0.47–2.12)	1.0	1.31 (0.84–2.04)
HR model 3^d^	1.0	0.98 (0.46–2.09)	1.0	1.27 (0.82–1.98)
***Stroke*** (cases/person-years)	36/91 920	9/9 618	95/71 959	29/10 890
Crude rate per 1000 person-years	0.4	0.9	1.3	2.7
HR model 1^b^	1.0	1.27 (0.58–2.76)	1.0	1.60 (1.03–2.49)
HR model 2^c^	1.0	1.12 (0.51–2.46)	1.0	1.54 (0.98–2.43)
HR model 3^d^	1.0	1.10 (0.50–2.42)	1.0	1.51 (0.96–2.37)
***Cancer*** (cases/person-years)	341/91 920	43/9 618	366/71 959	96/10 890
Crude rate per 1000 person-years	3.7	4.5	5.1	8.8
HR model 1^b^	1.0	0.72 (0.52–1.00)	1.0	1.09 (0.86–1.38)
HR model 2^c^	1.0	0.68 (0.49–0.95)	1.0	1.08 (0.85–1.38)
HR model 3^e^	1.0	0.68 (0.49–0.95)	1.0	1.07 (0.84–1.37)

a Type 2 diabetes, myocardial infarction, stroke or cancer, whichever occurs first.

b Stratified by age and adjusted for sex.

c Additionally adjusted for sleeping disorders (yes/no), sleep duration at night (<6, 6-<7, 7-<8, 8-<9, ≥9 h), alcohol intake from beverages (non-consumers, men: >0–12 g/d, >12–24 g/d, >24 g/d; women: >0–6 g/d, >6–12 g/d, >12 g/d), smoking status (never, former, current), walking, cycling, sports (hours/week), employment status (employed vs. unemployed), and education (technical school or lower degree vs. university of applied sciences or university degree).

d Adjusted for the variables in model 2 plus BMI (kg/m2), waist-to-hip ratio, and history of high blood lipid levels at baseline (yes/no).

e Model 3 for cancer includes the same covariates as models for type 2 diabetes, myocardial infarction and stroke, except history of high blood lipid levels at baseline (yes/no).

## Discussion

### Short sleep and chronic diseases

Our results showed that, compared to average sleep (7-<8 h), short sleep (<6 h per day) was associated with a 30% increased risk of overall chronic disease, in particular stroke (2-fold increased risk) and overall cancer (more than 40% increased risk).

This analysis was based on a first incident event analysis which is a novel approach that enables to compare the effect of sleep on different endpoints such as diabetes, cardiovascular diseases, and cancer. There has been previous evidence that short sleep (<5–6 h) is a habit that is associated with increased risk of chronic diseases [3], [10], [11], [12], [13].

Many studies investigated the association between sleep duration and diabetes risk and some of them showed that short sleep is an independent risk factor for diabetes [14], [15], [16], [17], [18]. In contrast, our results showed no relation between short sleep duration and incidence of diabetes after adjustment for potential intermediates. Similar to us, Hayashino et al. [34] and Björkelund et al. [35] did not find a significant association of sleep duration and diabetes risk. A possible reason for this discrepancy is the role of BMI in the relationship of sleep duration and diabetes as a potential mediator. It is often described in the literature that sleep restriction may lead to obesity by a number of biological mechanisms, e.g. via increased insulin resistance or via altered secretion of hormones like ghrelin or cortisol, which in turn could lead to subsequent diabetes [3], [10], [36], [37]. Thus, our risk estimates for short sleep duration as well as those of Ayas et al. [25] were attenuated to non-significant risk estimates after adjustment for BMI. In addition, hypertension [38] is a further potential intermediate that contribute to attenuation of the association between sleep duration and diabetes.

Fewer studies were conducted on sleep deficiency in relation to stroke and myocardial infarction. Chen et al. reported associations of short sleep duration with ischemic stroke in postmenopausal women (HR = 1.14, 95% CI: 0.97–1.33 for ≤6 h compared to 7 h of sleep), which became significant after exclusion of those with baseline comorbidities like cardiovascular disease or diabetes mellitus (HR = 1.22, 95% CI: 1.03–1.44) [21]. In contrast, Amagai et al. found no association of sleep duration and stroke after multivariable adjustment [22] which might be due to a relative small number of cases incorporated into this analysis. Regarding myocardial infarction we could not identify a significant relation with short sleep duration which is in contrast to the results of some previous studies, e.g. the Monitoring Trends and Determinants on Cardiovascular disease (MONICA) study [20], or the Japanese Jichi Medical Cohort Study [22], that reported on increased risk of myocardial infarction among women or men with short sleep. In our analyses, the association of sleep duration and myocardial infarction was significant in the sex- and age-adjusted models but inclusion of further covariates, especially smoking, but also alcohol drinking, hypertension, and satisfaction with health, removed the effect of sleep.

To our knowledge, this is the first study analyzing the relationship between sleep duration and overall cancer risk thereby observing an increased risk of developing cancer among participants reporting <6 h of sleep. Only a few studies examined the association between short sleep duration and breast cancer showing varying results. Kakizaki et al. found that women with ≤6 h sleep are at increased risk of developing incident breast cancer (HR = 1.62, 95% CI: 1.05–2.50; *p* for trend = 0.03) [24] whereas results by other researchers indicate that a long sleep duration of 9 h or more might have protective effects for breast cancer risk [39], [40]. On the contrary, data from the Nurses' Health Study does not support an inverse association of long sleep duration and breast cancer risk [41]. Furthermore, these authors called upon more research on the specific cancer sites and their relation to sleep quantity or quality.

### Biological mechanisms

A number of possible explanations for the association between decreased sleep duration and chronic diseases can be discussed. Short-term sleep deprivation induces inflammatory processes as well as an activation of the sympathetic nervous system, increased blood pressure, higher evening cortisol levels, and impaired glucose tolerance [42], [43], [44], [45], [46], [47] thus leading to an elevated risk of cardiovascular diseases and diabetes. Moreover, short sleep was shown to be related to increased levels of ghrelin, an appetite stimulant, and decreased levels of leptin, a satiety factor, which promotes appetite and food intake thereby leading to an increased risk of obesity [10]. However, total energy intake or food intake did not substantially differ across sleep categories in this cohort (see **Table 1**). More research is necessary to examine the relationship between sleep duration and food intake or meal patterns and meals contents.

Concerning cancer, current experimental and epidemiological evidence supports a link between melatonin production and risk of the development of cancer, especially breast cancer. Melatonin is a pineal hormone that is involved in the circadian regulation and facilitation of sleep, the inhibition of cancer development and growth as well as the enhancement of immune function. Individuals with short night sleep experience a suppression of melatonin secretion. Thus, these individuals are more likely to suffer from sleep disturbances, immune suppression and moreover are at a higher risk of developing a number of different cancers [11]. In addition, other mechanisms like inflammatory processes or changes in the autonomous tone could possibly mediate the link between sleep deficiency and cancer risk. Still, further research is necessary to get advanced insight into the relationship between sleep deficiency and cancer.

### Long sleep and chronic diseases

In agreement with others, our study indicates that prolongation of sleep above 9 h might be also not the best option. We found that risk of stroke was also increased by 65% in persons reporting long sleep duration (≥9 h). Similar observations were made by Chen et al. who found not only short sleep but also long sleep duration to be associated with ischemic stroke in postmenopausal women (HR = 1.24, 95% CI: 1.04–1.47 and HR = 1.70, 95% CI: 1.32–2.21 for women reporting 8 and ≥9 h of sleep) [21]. In addition, an increased risk of stroke was observed only for those sleeping more than 8 h in the First National Health and Nutrition Examination Survey (NHANES-I) [27]. Previous studies suggest that long sleep is further associated with an increased risk of developing incident diabetes [15], [16], [25], [26] which was not confirmed by our study. Of note, these studies were less stringent in excluding participants with chronic diseases at baseline which could affect sleeping hours.

The association of long sleep with cardiovascular diseases like stroke could be explained by poor sleep quality or poor health at baseline, e.g. chronic inflammation, unknown sleep apnea that could lead to subsequent hypertension, or circulatory disturbances in the brain, factors for which we could not control for in the statistical analysis. Long sleep may therefore represent an epiphenomenon of co-morbidity [48]. The positive association of a long overall sleep duration per day with stroke is also reflected by the association of daytime sleep and stroke among hypertensives which might be responsible for this overall effect.

### Daytime naps and chronic diseases

It is important to note that there had been effect modification by prevalent hypertension at baseline regarding daytime sleep. Among hypertensive persons reporting daytime naps the risk for overall chronic disease, in particular stroke, tend to be increased; whereas among non-hypertensive participants that stated to sleep during the day the risk of overall chronic disease, primarily cancer, is decreased.

This observation could be explained by a worse health condition or relevant symptomatic sleep-related breathing disorder (SRBD) among persons with hypertension reporting daytime sleep. It has been shown that persons with poor health, pain, depression, or those with poorer quality of sleep at night, e.g. by fragmented sleep with elevated number of arousals, in general tend to have daytime naps more frequently [49], [50], [51]. Therefore taking long naps was found to be associated with higher morbidity and mortality, especially among the elderly [17], [28], [52], [53], which is in line with our findings.

In contrast, short daytime naps among healthy persons might be a part of good lifestyle or a result of good sleep hygiene with stress relieving effects like it is making siesta in southern Europe [52], [54], [55]. A nap of less than 30 min duration during the day promotes wakefulness and alertness, reduces a sleep deficit and enhances performance and learning ability. Thus, daytime sleeping habits in general can reflect different concepts of lifestyle and health.

### Strengths and limitations of the EPIC-Potsdam study

The present study has a number of strengths including its prospective nature, the large sample size, high rate of follow-up, and verification of the self-reported information on disease through medical reports [30].

Though, with a recruitment proportion of 22.7% of the originally inquired persons, the EPIC-Potsdam cohort does not present a representative sample of the general population. In most studies, hence also in EPIC-Potsdam, the participating people are more health-conscious than non-participating people. Comparison to data from a representative health survey in eastern Germany showed that study participants in EPIC-Potsdam are characterized by a higher socio-economic status, higher health consciousness, and a better health status than the general population in East Germany [56].

One important methodological limitation of this study is the fact that sleep duration was self-reported and measured only at study recruitment. The Coronary Artery Risk Development in Young Adults (CARDIA) study showed that subjective reports of habitual sleep may be biased by systematic over-reporting among participants with short sleep durations compared to actigraph-measured sleep [57]. However, other comparisons of subjective sleep duration to the gold standard, polysomnography, or other more direct assessments like actigraphy showed good correlations [58], [59].

To increase validity of the results, it would be preferable to rely on repeated objective assessment of sleep quantity and quality, such as actigraphy [60] or even polysomnography [61], although feasibility of such methods might be questionable in large-scale cohort studies.

Another drawback of the EPIC study is that information on shift work or individual sleep requirement is missing. Furthermore, our question about the presence of any sleep disorder treated by a physician can not be regarded as a valid medical diagnosis and might underestimate the total prevalence of sleep disturbances. We also do not have further information about sleep quality or specific sleep disorders, like for example insomnia, sleep-disordered breathing (snoring, sleep apnea), restless legs syndrome, hypersomnia, or parasomnia, which might mediate the relationship of sleep and chronic diseases.

A further limitation is that all types of cancer were combined to one endpoint due to small number of cases for single cancer sites in this sample. However, in view of public health messages it is most relevant to ask whether the overall risk of cancer is impacted by sleep deprivation, independent of the type of cancer.

### Conclusion

In conclusion, short sleep duration is a strong risk factor for development of chronic diseases and thus is of significant public health relevance as sleep habits are amenable to behavioral interventions. This may be especially important in the context of the reduced sleeping hours observed during the last decades. However, compared to other lifestyle factors, e. g. nutrition, smoking, or physical activity, sleep is less apparent to the public to be a modifiable risk factor for chronic diseases. Therefore, it is important to increase awareness of the importance of sleep among health professionals and the lay public. Since short sleep is not only a personal choice also the roots of sleep deprivation, for instance shift work and prolonged working hours, should be addressed by making changes in work patterns and access to sleep recovery [7], [8]. Future studies should explore the efficacy of sleep interventions or rather changes in sleep habits for prevention of chronic disease.

## Supporting Information

Table S1 **Sleep Duration at Night and Risk of Chronic Diseases.** ^a^ Type 2 diabetes, myocardial infarction, stroke or cancer, whichever occurs first. ^b^ Stratified by age and adjusted for sex. ^c^ 95% confidence intervals are presented in parentheses. ^d^ Additionally adjusted for sleeping disorders (yes/no), alcohol intake from beverages (non-consumers, men: >0–12 g/d, >12–24 g/d, >24 g/d; women: >0–6 g/d, >6–12 g/d, >12 g/d), smoking status (never, former, current), walking, cycling, sports (hours/week), employment status (employed vs. unemployed), and education (technical school or lower degree vs. university of applied sciences or university degree). ^e^ Adjusted for the variables in model 2 plus sleep during the day (in hours) and potential intermediates: BMI (in kg/m^2^), waist-to-hip ratio, prevalent hypertension at baseline (yes/no) and history of high blood lipid levels at baseline (yes/no). ^f^ Model 3 for cancer includes the same covariates as models for type 2 diabetes, myocardial infarction and stroke, except prevalent hypertension at baseline (yes/no), history of high blood lipid levels at baseline (yes/no).
(DOC)
