# Supplementary material for: Association of Sleep Duration with Chronic Diseases in the European Prospective Investigation into Cancer and Nutrition (EPIC)-Potsdam Study
Source: PLoS One. 2012 Jan 25;7(1):e30972. doi: 10.1371/journal.pone.0030972 (PMC3266295; doi:10.1371/journal.pone.0030972)
Supplement: Table S1 — Sleep Duration at Night and Risk of Chronic Diseases. a Type 2 diabetes, myocardial infarction, stroke or cancer, whichever occurs first. b Stratified by age and adjusted for sex. c 95% confidence intervals are presented in parentheses. d Additionally adjusted for sleeping disorders (yes/no), alcohol intake from beverages (non-consumers, men: >0–12 g/d, >12–24 g/d, >24 g/d; women: >0–6 g/d, >6–12 g/d, >12 g/d), smoking status (never, former, current), walking, cycling, sports (hours/week), employment status (employed vs. unemployed), and education (technical school or lower degree vs. university of applied sciences or university degree). e Adjusted for the variables in model 2 plus sleep during the day (in hours) and potential intermediates: BMI (in kg/m2), waist-to-hip ratio, prevalent hypertension at baseline (yes/no) and history of high blood lipid levels at baseline (yes/no). f Model 3 for cancer includes the same covariates as models for type 2 diabetes, myocardial infarction and stroke, except prevalent hypertension at baseline (yes/no), history of high blood lipid levels at baseline (yes/no). (DOC) [file pone.0030972.s001.doc]

Table S1 Sleep Duration at Night and Risk of Chronic Diseases

|  | **<6 h/night** | **6- <7 h/night** | **7- <8 h/night** | **8- <9 h/night** | **≥9 h/night** |
| --- | --- | --- | --- | --- | --- |
| N total = 23 620 | 1 418 | 4 946 | 10 029 | 6 108 | 1 119 |
| Overall chronic disease (cases/person-years) ^a^ | 183 / 10 663 | 423 / 38 173 | 808 / 79 866 | 519 / 47 265 | 109 / 8 420 |
| Crude rate per 1000 PY | 17.2 | 11.1 | 10.1 | 11.0 | 12.9 |
| HR model 1 ^b^ | 1.49 (1.27-1.75)^c^ | 1.06 (0.94-1.19) | 1.0 | 1.03 (0.92-1.15) | 0.98 (0.80-1.21) |
| HR multivariable-adjusted, model 2 ^d^ | 1.41 (1.20-1.66) | 1.04 (0.92-1.17) | 1.0 | 1.01 (0.91-1.13) | 0.93 (0.76-1.14) |
| HR multivariable-adjusted, model 3 ^e^ | 1.27 (1.08-1.50) | 1.00 (0.89-1.12) | 1.0 | 0.99 (0.88-1.11) | 0.90 (0.73-1.10) |
|  |  |  |  |  |  |
| Type 2 diabetes (cases/person-years) | 73 / 10 663 | 182 / 38 173 | 331 / 79 866 | 210 / 47 265 | 45 / 8 420 |
| Crude rate per 1000 PY | 6.8 | 4.8 | 4.1 | 4.4 | 5.3 |
| HR model 1 ^b^ | 1.48 (1.14-1.90) | 1.11 (0.92-1.33) | 1.0 | 1.05 (0.88-1.25) | 1.06 (0.77-1.46) |
| HR multivariable-adjusted, model 2 ^d^ | 1.40 (1.08-1.80) | 1.09 (0.91-1.30) | 1.0 | 1.03 (0.86-1.22) | 1.00 (0.73-1.37) |
| HR multivariable-adjusted, model 3 ^e^ | 1.07 (0.82-1.39) | 0.99 (0.82-1.19) | 1.0 | 0.99 (0.83-1.18) | 0.92 (0.67-1.26) |
|  |  |  |  |  |  |
| Myocardial infarction (cases/person-years) | 20 / 10 663 | 38 / 38 173 | 83 / 79 866 | 47 / 47 265 | 9 / 8 420 |
| Crude rate per 1000 PY | 1.9 | 1.0 | 1.0 | 1.0 | 1.1 |
| HR model 1 ^b^ | 1.66 (1.02-2.72) | 0.95 (0.65-1.39) | 1.0 | 0.97 (0.67-1.39) | 0.87 (0.43-1.75) |
| HR multivariable-adjusted, model 2 ^d^ | 1.42 (0.87-2.33) | 0.86 (0.57-1.27) | 1.0 | 0.92 (0.64-1.32) | 0.74 (0.37-1.50) |
| HR multivariable-adjusted, model 3 ^e^ | 1.33 (0.80-2.22) | 0.84 (0.57-1.23) | 1.0 | 0.89 (0.62-1.28) | 0.70 (0.35-1.42) |
|  |  |  |  |  |  |
| Stroke (cases/person-years) | 18 / 10 663 | 31 / 38 173 | 63 / 79 866 | 41 / 47 265 | 16 / 8 420 |
| Crude rate per 1000 PY | 1.7 | 0.8 | 0.8 | 0.9 | 1.9 |
| HR model 1 ^b^ | 1.86 (1.10-3.14) | 1.01 (0.66-1.55) | 1.0 | 1.03 (0.69-1.53) | 1.79 (1.02-3.14) |
| HR multivariable-adjusted, model 2 ^d^ | 1.76 (1.03-3.00) | 1.00 (0.65-1.54) | 1.0 | 1.00 (0.67-1.48) | 1.64 (0.93-2.89) |
| HR multivariable-adjusted, model 3 ^e^ | 1.66 (0.97-2.86) | 0.98 (0.64-1.51) | 1.0 | 0.96 (0.65-1.43) | 1.56 (0.89-2.75) |
|  |  |  |  |  |  |
| Cancer (cases/person-years) | 73 / 10 663 | 174 / 38 173 | 336 / 79 866 | 224 / 47 265 | 39 / 8 420 |
| Crude rate per 1000 PY | 6.8 | 4.6 | 4.2 | 4.7 | 4.6 |
| HR model 1 ^b^ | 1.38 (1.07-1.78) | 1.05 (0.87-1.26) | 1.0 | 1.03 (0.87-1.22) | 0.78 (0.56-1.10) |
| HR multivariable-adjusted, model 2 ^d^ | 1.35 (1.04-1.74) | 1.03 (0.86-1.24) | 1.0 | 1.02 (0.86-1.21) | 0.77 (0.55-1.08) |
| HR multivariable-adjusted, model 3 ^f^ | 1.38 (1.06-1.78) | 1.04 (0.86-1.24) | 1.0 | 1.02 (0.86-1.21) | 0.78 (0.55-1.09) |

^a^ Type 2 diabetes, myocardial infarction, stroke or cancer, whichever occurs first.

^b^ Stratified by age and adjusted for sex.

^c^ 95 % confidence intervals are presented in parentheses.

^d^ Additionally adjusted for sleeping disorders (yes/no), alcohol intake from beverages (non-consumers, men: >0‑12 g/d, >12-24 g/d, >24 g/d; women: >0-6 g/d, >6-12 g/d, >12 g/d), smoking status (never, former, current), walking, cycling, sports (hours/week), employment status (employed vs. unemployed), and education (technical school or lower degree vs. university of applied sciences or university degree).

^e^ Adjusted for the variables in model 2 plus sleep during the day (in hours) and potential intermediates: BMI (in kg/m^2^), waist-to-hip ratio, prevalent hypertension at baseline (yes/no) and history of high blood lipid levels at baseline (yes/no).

^f^ Model 3 for cancer includes the same covariates as models for type 2 diabetes, myocardial infarction and stroke, except prevalent hypertension at baseline (yes/no), history of high blood lipid levels at baseline (yes/no).
